# Return to pregnancy after contraceptive discontinuation to become pregnant: a pooled analysis of West and East African populations

**DOI:** 10.1186/s12978-021-01193-w

**Published:** 2021-07-02

**Authors:** Janine Barden-O’Fallon, Ilene S. Speizer, Lisa M. Calhoun, Nouhou Abdoul Moumouni

**Affiliations:** 1 Department of Maternal & Child Health, Gillings School of Global Public Health, University of North Carolina, Chapel Hill, USA; 2 Carolina Population Center, University of North Carolina, Chapel Hill, USA; 3 L’Initiative OASIS, Niamey, Niger

**Keywords:** Contraceptive discontinuation, Return to pregnancy, Fecundity, Youth, Sub-Saharan Africa, West Africa, East Africa

## Abstract

**Background:**

The fear of infertility or delayed return to fertility is a common barrier to contraceptive use in sub-Saharan Africa, particularly among young or nulliparous women. Global evidence on return to pregnancy after method discontinuation suggests these fears may be misplaced; yet the topic has not been widely studied in sub-Saharan Africa nor by age and parity group.

**Methods:**

Reproductive calendar data from recent Demographic and Health Surveys of 15 sub-Saharan African countries were used to analyze time-to-pregnancy following discontinuation of a contraceptive method with the reason to become pregnant. The probability of pregnancy at 12 months was estimated using single-decrement life tables run by type of method discontinued, age and parity. Results are presented by region: francophone West Africa, anglophone West Africa and East Africa.

**Results:**

The 12-month probability of pregnancy after discontinuation of contraception to become pregnant was 73.0% in francophone West Africa, 78.8% in anglophone West Africa, and 82.0% in East Africa. Our results showed significant regional differences in return to pregnancy by 12 months, with probabilities in francophone West Africa being significantly lower than in anglophone West Africa or East Africa. A lower return to pregnancy by 12 months was seen among women ages 35–49 years and was lowest after discontinuation of a hormonal method for all age groups. Differences by parity group were only evident after discontinuation of hormonal methods in francophone West Africa.

**Conclusions:**

Sustainable gains in increasing contraceptive uptake, especially among youth, may be difficult to achieve without information and counseling that address concerns about infertility and potential delays in return to pregnancy following use of hormonal methods.

## Plain English summary

The fear of infertility or delayed return to fertility is a common barrier to contraceptive use in sub-Saharan Africa, particularly among young or childless women. Global evidence on the time it takes to become pregnant after the discontinuation of a contraceptive method suggests these fears may be misplaced; yet the topic has not been widely studied in sub-Saharan Africa. Data from recent Demographic and Health Surveys of 15 sub-Saharan African countries were used to analyze the time it takes to become pregnant after discontinuation of a contraceptive method with the reason to become pregnant. The probability of becoming pregnant by 12 months was estimated using a life table approach. Results were compared by type of method discontinued, age and whether women had any children, in three regions of sub-Saharan Africa. Our findings show that the 12-month probability of pregnancy after discontinuation of contraception to become pregnant was 73.0% in francophone West Africa, 78.8% in anglophone West Africa, and 82.0% in East Africa. A lower return to pregnancy by 12 months was seen among women ages 35–49 years and was lowest after discontinuation of a hormonal method for all age groups. Differences by whether women had any children were only evident after discontinuation of hormonal methods in francophone West Africa. The findings indicate that sustainable gains in increasing contraceptive uptake, especially among youth, may be difficult to achieve without information and counseling that address concerns about infertility and potential delays in return to pregnancy following use of hormonal methods.

## Background

Family planning is an essential maternal and child health service that has been offered in the African context for over 50 years. Despite much programmatic success throughout the continent, modern contraceptive use continues to be relatively low in sub-Saharan Africa, with a median level of use of 18.4% for 2000–2015 compared to 60.9% for Asia, and 66.7% for Latin America and the Caribbean during the same period [1]. Unmet need for family planning remains high in Africa at 23.5% of married women [2]. Individuals and couples in the region face a number of barriers to the adoption and continued use of contraception, including lack of access to high quality contraceptive information, services, and methods; a social context that highly values childbearing; and significant fear of side effects and health effects of using a method [2–6]. Among youth in sub-Saharan Africa, barriers to contraceptive use also include a lack of knowledge about contraceptive methods and services; the costs of obtaining family planning services; laws and policies that restrict access to young people; provider restrictions for provision of contraception to young people; fears, embarrassment and shyness around the topic of family planning; and social pressures to bear a child soon after marriage [7–11].

One of the key fears youth have about contraceptive use is the fear of infertility and concerns about a delayed return to fertility, especially following hormonal contraceptive use [6, 12–17]. Qualitative research from across sub-Saharan Africa finds that the fear of infertility is a frequently mentioned reason for non-use of contraception: this was found among urban youth in Mali and urban and peri-urban youth in Kenya [13, 15]. In addition, a study from Malawi demonstrated that among young women who perceived themselves as potentially infertile, one of the common reasons for this infertility perception was related to their past or present use of contraception [18]. The expression of fertility concerns as a barrier to method uptake can be linked to widespread stigma associated with infertility and the consequential high individual, family and social costs of infertility [19]. This stigma can greatly influence reproductive decision-making, as evidenced by a study among youth in Benin City, Nigeria, which found that youth expressed a preference for induced abortion over the potential for future infertility caused by contraceptive use [16]. The fear of infertility may also lead to low method satisfaction and early discontinuation [20]. This fear can also lead healthcare providers, parents, and family members to guide young people (and/or nulliparous women) away from the use of modern contraception [21].

Despite these fears, the prevalence of infertility in the sub-Saharan Africa region has declined; primary infertility (the inability to attain a live birth) among child-seeking women aged 20–44 fell from 2.7% in 1990 to 1.9% in 2010, equaling the global average [22]. Additionally, studies that compared the return to fertility after discontinuation of different contraceptive methods show minimal differences. A recent pooled analysis of 14,884 women from 22 studies published between 1985 and 2017 found a rate of pregnancy return of 83.1% (95% CI 78.2–88.0) within the first 12 months of discontinuation of a reversible contraceptive method [23]. The review found higher levels of pregnancy in the year after discontinuation of a non-hormonal method in many studies, but levels of pregnancy after discontinuation of hormonal methods compared to after discontinuation of non-hormonal methods were not statistically different [23]. The effects of age and parity were inconclusive across the studies [23]. A previously published systematic review of literature through 2009 found similar results: of the 17 prospective clinical and observational studies included in the review, the 12-month pregnancy rates following discontinuation of any method ranged between 71 and 96% across methods and studies [24]. The authors noted that pregnancy rates after discontinuation of a hormonal method were “broadly similar” to after discontinuation of barrier methods [24 p. 465]. In part due to small sample sizes, the effects of age and parity were non-conclusive. The authors concluded that the “past use of any contraceptive method does not reduce long-term subsequent fertility and that concern for subsequent fertility should not influence contraceptive decision making” [24 p. 470]. It is notable that only one of the studies included in both systematic reviews was located in the African continent, a prospective observational study in Ethiopia with 780 participants following discontinuation of the copper-IUD [25]. Furthermore, in the more recent study, only a small number of women in the pooled sample discontinued injectable contraception (n = 139 out of 14,884 discontinuers), meaning that the results are not necessarily informative for the sub-Saharan context, where injectable contraception is one of the most commonly used spacing methods [23].

The WHO Medical Eligibility Criteria provide evidence-based information and guidance to ensure an informed, voluntary choice of contraceptive methods, including information on return to fertility after method discontinuation [26]. The WHO guidance states that there is “…prompt return to fertility upon method discontinuation, with the exception of depot medroxyprogesterone acetate (DMPA) and norethisterone enanthate (NET-EN). The median delay in return to fertility with these methods is 10 and 6 months, respectively, from the date of the last injection, regardless of the duration of their use” [26, p. 104]. As a result of a lack of research on the effects of age and parity, the guidance is the same whether potential method users are young and/or nulliparous.

The objective of this study is to contribute evidence on return to pregnancy after contraceptive use, particularly hormonal contraceptive use, that fills gaps in prior work by focusing on sub-Saharan Africa with a particular focus on the West and East African regions. The francophone West African region was initially selected for analysis due to its low level of contraceptive use and strong socio-cultural norms to conceive which lead to normative barriers against family planning. A comparison with the anglophone West Africa and East African regions was added, as countries in these regions have comparably higher rates of contraceptive use, including injectable use, and acceptance of family planning. This research addresses whether there are significant, notable differences in the 12-month probability of pregnancy after discontinuation of a method for adolescents and youth and for women at zero parity. The research compares the pregnancy probabilities for women who discontinued a method because they wanted to get pregnant between francophone West Africa (FWA), anglophone West Africa (AWA), and East Africa (EA) by type of hormonal method used, age group, and parity group. The results can be used to contribute to training of providers on method counseling for youth and broader community-based activities in these regions where concerns about infertility or delayed return to fertility are common reasons for non-use and where providers may not recommend hormonal methods to young women.

## Methods

Data for this analysis came from the Demographic and Health Survey (DHS). The DHS is a nationally representative household survey that provides data on a wide number of population and health outcomes, including family planning. The DHS uses a stratified two-stage sampling design composed of randomly selected households within primary sampling units. Detailed information on the design and sampling, as well as the standardized survey questionnaires used by the DHS, can be found at https://dhsprogram.com/What-We-Do/Survey-Types/DHS-Methodology.cfm. All DHS receive appropriate ethical approvals prior to country implementation. Additionally, all respondents voluntarily consent to participate in the survey. The DHS data are publicly available for research in an anonymous format. This secondary analysis study was assessed by the University of North Carolina Institutional Review Board and determined exempt from further review (Study #19-2251).

One of the instruments often included in the DHS survey is a reproductive (or “contraceptive”) calendar, which is a month-by-month retrospective history of contraceptive use, non-use, pregnancies, terminations, and live births occurring during at least 5 calendar years preceding the survey interview. The calendar is applied to women of reproductive age, typically ages 15–49. Calendar data are particularly useful to study sequential reproductive events and have been used to examine the reproductive consequences of method failure, patterns of method discontinuation and switching, and the contribution of contraceptive discontinuation to unintended births, to name but a few [27–30]. Countries were included in the analysis if they (1) were located in the FWA, AWA or EA regions and (2) had calendar data from a recent DHS (2010 or later). The countries meeting these criteria and included in the analysis were: Benin (2017–2018), Burkina Faso (2010), Guinea (2018), Mali (2018), Niger (2012), and Senegal (2017) for the FWA region; Gambia (2013), Ghana (2014), Liberia (2013), Nigeria (2018), and Sierra Leone (2013) for the AWA region; and Ethiopia (2016), Kenya (2014), Tanzania (2015–2016), and Uganda (2016) for the EA region. Some of the reproductive calendars included information for periods longer than 5 years, this analysis focuses on the 60 months before the survey for standardization across countries. Country data were pooled for analysis by region.

Contraceptive methods included in the calendar are female and male sterilization, IUD, injectables, implants, pill, condom, female condom, emergency contraception, Standard Days Method, lactational amenorrhea method, rhythm method, withdrawal, “other modern method”, and “other traditional method”. Only one method is recorded for each month. The calendar data do not differentiate between type of injectable (intramuscular or subcutaneous) or type of IUD (copper-containing or Levonorgestrel-releasing); during the study period, most of the injectable use was intramuscular and most IUD use was the copper IUD. For each reported method discontinuation, a reason is recorded. Reasons for method discontinuation include infrequent sex/husband away, became pregnant while using (i.e., method failure), wanted to become pregnant, husband/partner disapproved, wanted more effective method, side effects/health concerns, lack of access/too far, costs too much, inconvenient to use, up to God/fatalistic, difficult to get pregnant/menopausal, marital dissolution/separation, “other”, and don’t know.

The analysis of the calendar data was episode-based. An episode was defined as an uninterrupted period of time (measured in months) that began with one event, such as contraceptive use, nonuse, or pregnancy, and ended with a change to another event. Time-to-pregnancy episodes were defined as episodes that began with a contraceptive discontinuation for the reason of becoming pregnant and ended with a pregnancy or censoring at the time of the survey. A woman may contribute more than one such episode during the 5-year calendar period. Single-decrement life tables were used to estimate the 12-month probability of pregnancy. Time-to-pregnancy episodes were considered lost to follow-up if ending in re-uptake of a contraceptive method or if censured at the time of the survey. Time-to-pregnancy episodes beginning within 3 months prior to the survey were excluded from life table analysis to avoid the issue of under-reporting of pregnancies during the first trimester. Life tables were run by discontinued method type: “hormonal” (pill, injectable, implant) vs. “non-hormonal” (all other methods), by age at the time of the discontinuation event (ages 15–24, 25–34, and 35–49) and by parity at the time of the discontinuation event (nulliparous vs. primiparous or multiparous). Overall, IUD use is low across the study countries. As mentioned earlier, most IUD use in sub-Saharan Africa was the copper-IUD, and thus those women who reported discontinuing an IUD were included in the non-hormonal method category. Twelve-month pregnancy probabilities are shown with 95% confidence intervals. Life tables were also run for time-to-pregnancy episodes following discontinuation of each of the three hormonal methods. Kaplan Meier graphs show the method-specific survivor functions. Survivor functions were assessed using log-rank test for equality (χ^2^ statistic).

## Results

As shown in Table 1, there were a total of 5361 contraceptive discontinuations with the reason to become pregnant in the six FWA countries; these accounted for 43.1% of all contraceptive discontinuations. The three most commonly discontinued methods in FWA women wanting to become pregnant were pills (32.1%), injectables (28.2%) and implants (11.0%). This corresponds to the main methods used in these countries. In the five AWA countries, there were 3403 contraceptive discontinuations in order to become pregnant (34.6% of all discontinuations). The three most common discontinued methods for women wanting to become pregnant in this region were injectables (29.5%), pills (21.1%) and withdrawal (12.6%). In the four EA countries, there were 6734 contraceptive discontinuations for the reason to become pregnant (34.4% of all discontinuations). The three most common discontinued methods for women wanting to become pregnant in this region were injectables (60.3%), pills (15.1%) and implants (8.0%). A significantly lower percentage of discontinuations for the reason of becoming pregnant were among the oldest women as compared to the younger age categories. A higher percentage of discontinuations for the reason of becoming pregnant were also seen among women with one or more children as compared to women with no children in FWA and AWA, while the opposite was true in EA. Differences by age group and parity group were statistically significant at p < 0.05.

**Table 1:** Discontinuations to become pregnant by age and parity of woman and methods discontinued, by region

	Francophone West Africa N = 5361	Anglophone West Africa N = 3403	East Africa N = 6734
Discontinuations to become pregnant	n (% of all reasons)	n (% of all reasons)	n (% of all reasons)
Age (years)			
15–24	2175 (44.4)*	1120 (31.9)*	3186 (39.2)*
25–34	2569 (46.5)	1872 (40.5)	2986 (35.2)
35–49	617 (30.4)	411 (24.1)	562 (18.9)
Parity			
0 child	749 (37.5)*	572 (29.2)*	1210 (36.9)*
1+ child	4612 (44.2)	2831 (35.9)	5524 (33.9)

Method discontinued to become pregnant	n (%)	n (%)	n (%)
Pill	1723 (32.1)	718 (21.1)	1017 (15.1)
Injectable	1510 (28.2)	1004 (29.5)	4063 (60.3)
Implant	591 (11.0)	144 (4.2)	541 (8.0)
Condom	282 (5.3)	328 (9.6)	304 (4.5)
IUD	115 (2.1)	72 (2.1)	113 (1.7)
Lactational amenorrhea method	432 (8.1)	229 (6.7)	80 (1.2)
Standard days method	66 (1.2)	10 (0.3)	24 (0.4)
Other modern^a^	27 (0.5)	51 (1.5)	19 (0.3)
Periodic abstinence/rhythm	297 (5.5)	321 (9.4)	335 (5.0)
Withdrawal	147 (2.7)	428 (12.6)	202 (3.0)
Other traditional methods	169 (3.2)	98 (2.9)	36 (0.5)

^a^Includes female condoms, emergency contraception, and “other modern methods.”

*p < 0.05 demonstrating significant difference in distribution by age and parity

The 12-month probability of pregnancy after discontinuation by method type and region for the 14,496 discontinuation events with data on age and parity are shown in Table 2. The table shows that probabilities of pregnancy after all-method discontinuation ranged from 73.0% (CI 71.7–74.3%) in FWA to 82.0% (CI 81.0–82.9%) in EA; there was a general pattern of lower levels of pregnancy at 12 months after discontinuation in FWA compared to AWA or EA. Statistically significant (i.e., non-overlapping confidence intervals) lower probabilities of pregnancy were seen after discontinuation of hormonal methods as compared to non-hormonal methods across all three regions; differences varied by 7.8 percentage points in FWA to 11.3 percentage points in EA.

**Table 2:** Twelve-month probability of pregnancy after discontinuation by method type and region

Region	All methods	Hormonal	Non-hormonal
	Ages 15–49	Ages 15–49	Ages 15–49
Francophone West Africa			
Percent	73.0	70.8	78.6
(CI)	(71.7–74.3)	(69.2–72.3)	(76.3–80.8)
N	4998	3542	1456
Anglophone West Africa			
Percent	78.8	74.2	84.2
(CI)	(77.2–80.2)	(72.0–76.3)	(82.2–86.1)
N	3145	1707	1438
East Africa			
Percent	82.0	80.1	91.4
(CI)	(81.0–82.9)	(79.0–81.2)	(89.5–93.0)
N	6353	5297	1056

*CI* 95% confidence interval

For discontinuation of all methods by age group, the three regions showed a slower return to pregnancy in the 35–49 age group, with FWA having the lowest 12-month probability of pregnancy for this age group, at 67.2% (CI 63.2–71.1) (Table 3). There was no significant difference in pregnancy probabilities between age groups 15–24 and 25–34. However, differences in pregnancy probabilities after discontinuation were again evident by method type: lower pregnancy probabilities were seen for all age groups after discontinuation of hormonal methods as compared to discontinuation of non-hormonal methods. After discontinuation of all methods, parity was only significant in FWA, with a 12-month probability of pregnancy of 68.0% (64.4–71.6) for nulliparous women compared to 73.9% (72.5–75.2) for women with one or more children (Table 4). However, lower pregnancy probabilities were seen after discontinuation of hormonal methods in nulliparous women and women with one or more children as compared to their counterparts discontinuing non-hormonal methods in all regions.

**Table 3:** Twelve-month probability of pregnancy after discontinuation by method type, age group, and region

Region	All methods			Hormonal			Non-hormonal		
	Ages 15–24	Ages 25–34	Ages 35–49	Ages 15–24	Ages 25–34	Ages 35–49	Ages 15–24	Ages 25–34	Ages 35–49
Francophone West Africa									
Percent	72.9	74.6	67.2	70.8	71.7	66.5	77.7	82.3	69.2
(CI)	(70.9–74.9)	(72.7–76.4)	(63.2–71.1)	(68.3–73.3)	(69.4–73.9)	(61.7–71.3)	(74.2–81.0)	(79.1–85.3)	(62.2–76.0)
N	2029	2388	581	1404	1739	399	625	649	182
Anglophone West Africa									
Percent	77.8	80.6	73.1	72.7	76.2	69.4	84.7	85.4	77.4
(CI)	(75.1–80.4)	(78.6–82.5)	(68.3–77.7)	(68.9–76.4)	(73.2–79.0)	(62.6–76.0)	(81.0–88.0)	(82.7–87.8)	(70.7–83.5)
N	1046	1727	372	604	900	203	442	827	169
East Africa									
Percent	82.1	82.5	78.4	80.4	80.5	76.0	91.6	92.0	87.7
(CI)	(80.6–83.5)	(81.0–84.0)	(74.6–82.0)	(78.7–81.9)	(78.9–82.2)	(71.6–80.1)	(88.7–94.0)	(89.2–94.2)	(80.5–93.2)
N	3002	2825	526	2546	2335	416	456	490	110

*CI* 95% confidence interval

**Table 4:** Twelve-month probability of pregnancy after discontinuation by method type, parity group, and region

Region	All methods		Hormonal		Non-hormonal	
	0 child	1+ child	0 child	1+ child	0 child	1+ child
Francophone West Africa						
Percent	68.0	73.9	64.6	71.6	72.9	80.0
(CI)	(64.4–71.6)	(72.5–75.2)	(59.8–69.4)	(69.9–73.2)	(67.5–78.1)	(77.6–82.4)
N	704	4294	415	3127	289	1167
Anglophone West Africa						
Percent	80.4	78.4	74.0	74.2	85.3	83.9
(CI)	(76.7–83.8)	(76.8–80.1)	(67.9–79.7)	(71.8–76.5)	(80.9–89.2)	(81.6–86.1)
N	540	2605	239	1468	301	1137
East Africa						
Percent	82.0	82.0	79.2	80.3	90.3	91.7
(CI)	(79.6–84.3)	(80.9–83.0)	(76.3–82.0)	(79.0–81.5)	(86.3–93.6)	(89.6–93.6)
N	1125	5228	846	4451	279	777

*CI* 95% confidence interval

**Fig. 1:**
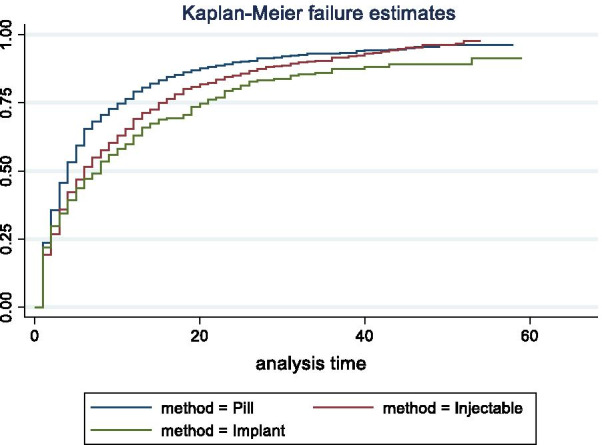
Cumulative pregnancy probabilities following discontinuation of pills, injectables, and implants, in Francophone West Africa

Cumulative pregnancy probabilities following discontinuation of pills, injectables, and implants are shown by Kaplan–Meier curves for the three regions in Figs. 1(FWA), 2(AWA) and 3(EA). In all three regions, the p-values indicate that at least one of the survivor functions is statistically different from the others. The return to pregnancy was highest following the discontinuation of pills, with 12-month pregnancy probabilities ranging from 77.4 to 85.7%, while probabilities after injectable and implant discontinuation were similar and range from 61.3 to 79.2% (Table 5). Overall, pregnancy probabilities for these three methods were lowest in the FWA region as compared to the other two regions.

**Fig. 2:**
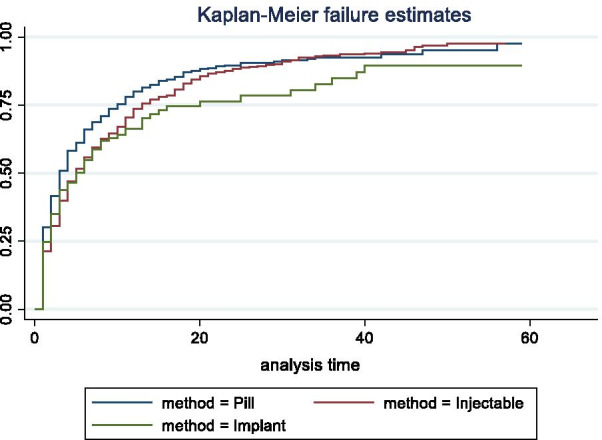
Cumulative pregnancy probabilities following discontinuation of pills, injectables, and implants, in Anglophone West Africa

**Fig. 3:**
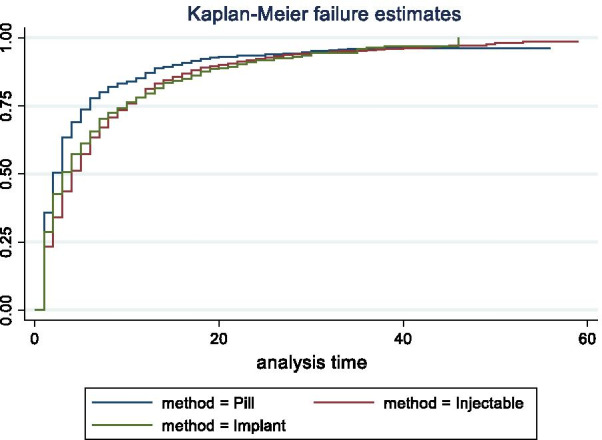
Cumulative pregnancy probabilities following discontinuation of pills, injectables, and implants, in East Africa

**Table 5:** Twelve-month probability of pregnancy after discontinuation of the pill, injectable or implant, by region

Region	Pill	Injectable	Implant
Francophone West Africa			
Percent	77.4	66.6	61.3
(CI)	(75.2–79.5)	(64.0–69.2)	(56.9–65.7)
N	1,616	1,396	530
Anglophone West Africa			
Percent	78.8	71.5	68.5
(CI)	(75.5–82.0)	(68.4–74.5)	(59.7–76.9)
N	670	912	125
East Africa			
Percent	85.7	78.8	79.2
(CI)	(83.4–87.9)	(77.4–80.1)	(75.3–82.9)
N	960	3,842	495

*CI* 95% confidence interval

## Discussion

In the sub-Saharan African context, where there are strong social pressures to get pregnant and demonstrate fecundability and strong pronatalist views, health concerns about infertility and contraceptive use, particularly for young women who have never had children, are often raised as reasons for non-use of contraception. While the Medical Eligibility Criteria do not recommend limitations to use of most of the common hormonal and long-acting methods (i.e., pills, injectables, implants, and IUDs) by age group or parity, provider biases often limit the offering of these methods to young women and nulliparous women [31]. Given concerns about fecundability post-contraceptive use, this analysis sought to examine the 12-month probability of pregnancy for women who used contraception but discontinued with a reason to get pregnant. The focus was on examining hormonal versus non-hormonal methods and on examining differences in 12-month pregnancy probabilities for young women (< 25), women ages 25–34, and older women (35+) and between nulliparous women versus women with one or more children. Comparisons were made by region and language group, comparing francophone West Africa to anglophone West Africa and East Africa.

These results revealed that two-fifths of method discontinuations in FWA were for the reason to become pregnant, while only one-third of discontinuations were for this reason in AWA and EA. The most common methods discontinued to become pregnant across all regions were injectables and pills; this is not surprising, given that pills and injectables are the most common methods used in sub-Saharan Africa. Furthermore, injectables and pills are both spacing methods, thus likely to be discontinued for the reason of wanting to become pregnant.

Our results demonstrated significant regional differences in the return to pregnancy by 12 months such that it was lower in FWA than the other two regions for all method types, age, and parity categories. Further, in all regions, the return to pregnancy was lower after use of hormonal methods compared to non-hormonal methods, likely driven by a slower return to fecundability after injectable use. Although fears of infertility and delayed return to fertility are often expressed by adolescents and youth, it was the oldest age group that showed the lowest pregnancy probabilities, supporting evidence that fecundability declines after peaking in the 20 s [32]. Differences by parity group were evident after discontinuation of hormonal and non-hormonal methods only in FWA, with the lowest probabilities among nulliparous women after hormonal contraceptive use.

Results presented for EA and AWA were consistent with those found in the 2018 Girum and Wasie systematic review, which found a 12-month pregnancy rate of 83.1% (95% CI 78.2–88.0) [23]; this estimate range is similar to our results despite the non-inclusion of the African context in the pooled analysis (with the exception of one study). Notably, as discussed here, the return to pregnancy in the FWA region is lower than in the other regions in our study, as well as lower than the Girum and Wasie (2018) pooled rate [23]. Our results also differ from previous analyses by showing that there were significant differences in return to pregnancy between hormonal and non-hormonal method users.

Understanding the root causes for differences in return to pregnancy across the three regions was beyond the scope of this analysis. As suggested by Mansour et al. [24], regional differences in behavioral, environmental, and genetic factors may affect fecundity and may be the key factor that affects time to pregnancy post-contraceptive use in the differing study regions. For example, in these study regions, there may be varying levels of latent infertility. There may also be variation in the motivation for pregnancy, reduction in sexual activity as women age, and/or variation in spousal residence patterns due in part to polygyny, which could affect the frequency of sexual intercourse and thus, exposure to becoming pregnant. Finally, cultural practices related to the duration of breastfeeding may also play a role in return to pregnancy for women discontinuing use of LAM, which was most common in FWA (8.1% of method discontinuations to become pregnant).

Although we are unable to explain why women from FWA have a lower pregnancy probability as compared to other regions, our findings do have programmatic implications. In particular, given that we show there is a longer return to pregnancy in FWA among hormonal method users and nulliparous women, it is perhaps not surprising that concerns about infertility and delayed return to fertility are a common reason for non-use. In this context, it continues to be important that providers, community health leaders, and others engaging with communities, understand that while return to pregnancy after hormonal contraceptive use is high, it may take longer than desired or expected for women living in FWA. Return to pregnancy should also be discussed in the context of other factors that affect fecundity, such as frequency of sexual intercourse related to partner separation or non-co-habitation. In these settings, informed counseling on return to fertility is important for all women adopting methods, and especially if hormonal methods are being offered. Without a clear understanding of return to fertility, dissatisfied users can influence social attitudes and subsequent adoption of the most-effective contraceptive methods. Further, for nulliparous women who are worried about future fertility or a delay in return to fecundability, it may be best to offer effective non-hormonal methods (e.g., condoms) or encourage pills over injectables or implants that take longer for return to fertility. In the AWA or EA context, a smaller percentage of discontinuations were for the reason of becoming pregnant, as were the differences in pregnancy probabilities after hormonal and non-hormonal method discontinuation. In these contexts, high-quality counseling may be enough to support continued use of the more effective methods for spacing and avoiding unintended pregnancies.

One of the strengths of this study is that it is one of the first studies to examine return to pregnancy using data from sub-Saharan Africa and comparing differences between the FWA, AWA, and the EA regions. In addition, by aggregating the samples across regions, it was possible to look at differences by age group (e.g., 15–24, 25–34, and 35+) and parity (0 prior birth vs. 1+ prior births). Different pregnancy probabilities by parity group and method choice provide important context for future program design and the strengthening of training for contraceptive counseling.

This study also has limitations. First, the contraceptive calendar may introduce recall bias if women are unable to remember exactly when reproductive events took place. While previous analysis of the calendar data demonstrates that the quality is sufficient to support analytical conclusions [33], more recent work suggests contraceptive use and discontinuation are under-reported and that under-reporting may be more likely the longer the recall period [34]. We used the standard period of 5 years for the calendar to maximize the amount of data available for the study, though experimentation with shorter periods is suggested as needing further investigation [34]. Second, in this analysis, discontinuation is self-reported. For some methods like the pill, implant or IUD, it is clear when discontinuation occurs (e.g., after stopping the pill or when the implant or IUD was removed). Conversely, for a method like the injection, women are self-reporting when they stopped use. They may report officially stopping after the 3-month recommended re-injection period or they may decide earlier (or later) that they are stopping use. This will affect the assessed time to pregnancy; for example, if women report discontinuation 1 month after the last injection, it will over-estimate the time to pregnancy. With the way that the contraceptive calendar is collected, there is no clear way to determine exactly when a woman “discontinues” injectable method use because there is no way to know when she received her last injection. Third, this analysis does not consider the outcome of pregnancy events. As a result, the analysis does not shed light on whether the method used before discontinuation affects whether pregnancies result in a live birth, miscarriage, or stillbirth. Finally, this analysis is premised on the assumption that women who report that they discontinue because they want to get pregnant are actually actively trying to get pregnant (i.e., their partner was present and they were seeking a pregnancy during the entire post-discontinuation period). While this may not be the case, we do not have additional data to help inform when the women are and are not actively seeking a pregnancy and therefore, all discontinuations due to wanting to become pregnant are treated as if pregnancy was actively being sought.

## Conclusion

The results of this study can be used to inform programs that offer family planning services in sub-Saharan Africa. Sustainable gains in increasing contraceptive uptake, especially among youth, may be difficult to achieve without information and counseling that addresses concerns about infertility and potential delays in return to fertility with some methods. In the FWA context, providers may prioritize offering pills or condoms over injectable contraception to nulliparous women who are concerned about infertility or delayed fertility, in order to avoid perceived delays in return to pregnancy that can lead to negative attitudes toward all method use. In all regions, service delivery approaches should ensure that women have access to full information about a full range of methods. “Full information” should include transparent information on average time to pregnancy. In the case of injectables (and implants), providers should follow WHO guidance and acknowledge that it can take longer to get pregnant upon discontinuation. This will help ensure that clients are making informed decisions prior to adopting these spacing (or delaying) methods.

## Data Availability

The DHS data analyzed for this study are publicly available for research in an anonymous format from The DHS Program at https://dhsprogram.com/data/.

## Abbreviations

AWA: Anglophone West Africa
DHS: Demographic and Health Survey
FWA: Francophone West Africa
